# Strong species-environment feedback shapes plant community assembly along environmental gradients

**DOI:** 10.1002/ece3.784

**Published:** 2013-09-20

**Authors:** Jiang Jiang, Donald L DeAngelis

**Affiliations:** 1 National Institute for Mathematical and Biological Synthesis, University of TennesseeKnoxville, Tennessee, 37996; 2 U.S. Geological Survey, Department of Biology, University of MiamiCoral Gables, Florida, 33124

**Keywords:** Alternative states, coexistence, community assembly, Ecosystem engineer, limiting similarity, neutral model, niche, species zonation

## Abstract

An aim of community ecology is to understand the patterns of competing species assembly along environmental gradients. All species interact with their environments. However, theories of community assembly have seldom taken into account the effects of species that are able to engineer the environment. In this modeling study, we integrate the species' engineering trait together with processes of immigration and local dispersal into a theory of community assembly. We quantify the species' engineering trait as the degree to which it can move the local environment away from its baseline state towards the optimum state of the species (species-environment feedback). We find that, in the presence of immigration from a regional pool, strong feedback can increase local species richness; however, in the absence of continual immigration, species richness is a declining function of the strength of species-environment feedback. This shift from a negative effect of engineering strength on species richness to a positive effect, as immigration rate increases, is clearer when there is spatial heterogeneity in the form of a gradient in environmental conditions than when the environment is homogeneous or it is randomly heterogeneous. Increasing the scale over which local dispersal occurs can facilitate species richness when there is no species-environment feedback or when the feedback is weak. However, increases in the spatial scale of dispersal can reduce species richness when the species-environment feedback is strong. These results expand the theoretical basis for understanding the effects of the strength of species-environment feedback on community assembly.

## Introduction

Niche-structured community assembly theory predicts that species distribute themselves according to their abiotic requirements and tolerances along abiotic environmental gradients, such as topography and edaphic characteristics (Pielou 1977). Nevertheless, recent studies highlight the role of dispersal, along with biotic interactions, both direct and indirect, in species distribution models (Boulangeat et al. 2012; Kissling et al. 2012). Dispersal limitation may restrict a species' range by preventing individuals from reaching suitable sites outside their current distribution, but species may also reach unsuitable sites and exhibit source-sink dynamics (Pulliam 2000). Biotic interactions include both direct competition and indirect effects of species, the latter through modifying either the resource availability or the local abiotic environment, which may result, alternatively, in either indirect competition with, or facilitation of other species (Callaway 1995; Bever 2003; Bruno et al. 2003).

The fact that species both affect and are affected by their environment has been integrated into contemporary niche theory (Chase and Leibold 2003). The term ‘ecosystem engineer’ has sometimes been applied to species that exert strong effects on their local environments (e.g., Jones et al. 1994, 1997; Odling-Smee et al. 2003). The term has also been criticized as being redundant because all species influence their environments (Reichman and Seabloom 2002). Thus, the importance of these engineering effects may be a matter of degree and spatial scale, as Hastings et al. (2007) suggested. However, until recently relatively little theoretical work has been done in exploring the consequences to community ecology of integrating species' engineering traits with local dispersal and immigration to the study of niche structured community assembly.

We quantify the species' engineering trait in terms of the strength of what we will term its ‘species-environment feedback’, or the magnitude of the effect the species has in altering its local environment. The question we address is whether the species-environment feedback, interacting with local dispersal and immigration, can explain the distribution of species of several vegetation types along such an environmental gradient. Vegetation crucially affects the morphodynamic processes configuring the elevation profiles of salt marshes. An example of such a mechanism is the ability of various marsh macrophytes to increase soil accretion rates and thus alter local elevation and flooding frequency (Morris 2006, 2007; Marani et al. 2006, 2013). Because different marsh vegetation types are found within narrow elevation bounds, the possibility has been suggested that particular species or groups of species can shape topography to maintain favorable environments for themselves (Silvestri et al. 2005; Mudd et al. 2010; D'Alpaos et al. 2012). Ability to affect local soil chemistry is another feedback mechanism that may affect zonation. The plant zonation along salinity gradients from seaward to inland is a case in point (Sternberg et al. 2007; Jiang et al. 2012a,b). Mangrove trees can cause accumulation of soil salinity through continued transpiration during the dry season, whereas freshwater plants can reduce transpiration as response to elevated salinity, thus preventing further salinity increase. The result of different species exerting different feedback on soil salinity may affect the patterns of both salinity and vegetation.

In this study, we explicitly address this question with a theoretical model, reflecting real examples such as the marsh macrophytes' ability to change soil morphology and mangroves' ability to change soil salinity. We investigate the case in which each species in the community tends to alter the baseline environment towards its own niche optimum, against the environment's tendency to recover towards its baseline state. Depending on the engineering strength of a species, the realized environment will move towards an intermediate state somewhere between the baseline state and that species' niche optimum. The environmental state altered by the engineer may facilitate some other species, if those species have higher fitness at the altered state than at the baseline state. We incorporate the degree of engineering effects by adjusting the relative strength of species-environment feedback to the recovery rate of the environment to its baseline state. We ask (1) how the species-environment feedback affects local diversity (we will use species richness as a representation of diversity here); (2) how the effect of increasing feedback strength on species richness (the diversity-feedback relationship) responds to different immigration rates and different scales of local dispersal; (3) how environmental heterogeneity impacts the interaction among the three factors; engineering strength, local dispersal, and immigration rate.

## Method

To be able to compare the present work with other theoretical work on community assembly, we followed similar modeling approaches used recently, which couple the lottery process of Hubbell's neutral model to niche differentiation in a spatially heterogeneous environment (Schwilk and Ackerly 2005; Gravel et al. 2006; Fukami and Nakajima 2011). The basic framework of the model is that the local patches are niche-structured with a continuous small input of individuals immigrating from a regional species pool. We assume the regional species pool has a fixed species number and pre-determined distribution not affected by feedback from the local patches. We simulated local patches using a simple one-dimensional lattice landscape with the goal of understanding the mechanisms driving community assembly along an environmental gradient.

Each cell on the lattice landscape can be inhabited by a single adult plant, and there is competition between plant species for occupation of the cell. The total number of individuals in the local landscape always equals the number of cells, *J*. At each time step, one plant is selected at random to die and be replaced by another plant either by local dispersal or immigration. For simplicity, we assume species have equal probabilities of mortality per unit time, which is the same assumption used in most of lottery models (Schwilk and Ackerly 2005; Gravel et al. 2006). However, recruitment probability of new adults of species *i* at location *x*, *R*_*i,x*_, equals immigration from the regional species pool plus weighted-lottery competition for that environment, which is a spatially explicit form taking into account both niche differentiation and dispersal processes


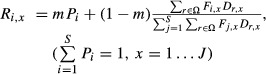
(1)

where parameter *m* is the probability that a recruit is immigrating from the regional species pool, weighted by its relative abundance *P*_*i*_ in the pool. The total number of species in the region's species pool is *S*. The fitness function (*F*_*i,x*_) for species *i* at location *x* is calculated from a Gaussian function, with a fundamental niche optimum at (*μ*_*i*_) and a fundamental niche breadth (*b*_*i*_).


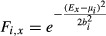
(2)

where *E*_*x*_ is the current value of environment at location *x*. The distribution of niche optima *μ*_*i*_ in the regional species pool is uniform across the range of available environments. The dispersal kernel (*D*_*r,x*_) is a Gaussian probability distribution centered at the parent plant's location, a distance *r* from the location *x*, with scale of dispersal *d*_*i*_. *r* is a given point in a space vector *Ω*, the Euclidean distance from a parent plant's location to location within the local landscape.


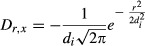
(3)

Species have the ‘engineering’ trait to alter abiotic local environment, the dynamics of which are described by the following equation,


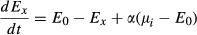
(4)

where *E*_*0*_ is the baseline state of the environment, which can be either uniformly distributed across space, or spatially varying, and where *α* is the relative strength of species-environment feedback to the recovery rate of the environment to its baseline state (0 ≤ α ≤ 1). Under positive feedback (0 < *α*), an ecosystem engineer drags its local environment towards its niche optimum (*μ*_*i*_), and precisely to *μ*_*i*_ when α = 1. Without feedback (*α* = 0) or in the absence of ecosystem engineers, the environment returns to its original baseline state *E*_*0*_, as described by eq. 4. The positive feedback here is assumed to maintain the species that creates the feedback. It may, by chance, facilitate other species if those species are better competitors at the equilibrium of the environment (Bertness and Callaway 1994), but it may also decrease the fitness of other species locally.

### Simulation

Our objective was to investigate impacts of species-environment feedback on community assembly along a landscape that is heterogeneous in the form of a linear gradient in baseline environmental conditions. However, for comparative purposes, we also considered a homogeneous landscape and one that is randomly heterogeneous (but to avoid complexity in the main text, this latter case is described in Appendix A). To do this we used a one-dimensional lattice landscape that could take any of these forms. The regional species pool was fixed, *S =* 100, with the species differing from each other only in their fundamental niche optima, *μ*_*i*_. Species niche optima were evenly distributed from 0 to 1.0. At the beginning of a simulation, the same number of initial species, *S* = 100, occupied the local landscape, with each lattice cell being occupied by a single adult of whichever species is the best competitor at the site of that cell.

On the one-dimensional landscape, each lattice cell was assigned a baseline environmental value, either homogeneously, with the same value, *E*_*0*_ *=* 0.5, for every lattice cell, *J =* 1000, or heterogeneously, in which *E*_*0*_ gradually increases linearly from 0 to 1.0 at intervals of 0.001 units, representing an underlying environmental gradient (the randomly heterogeneous landscape is described in Appendix A). We investigated three levels of immigration rate, or the probability that a recruit immigrates from the regional species pool at each time step; *m =* 0*,* 0.01 and 0.1. In case of *m =* 0, there were no additional immigrants from regional species pool after the initiation. We also explored four local dispersal scales, the standard deviation *d* in Gaussian distribution (eq. 3) in units of grid cells; *d =* 10, 20, 50 and 100. Niche breadth, *b* in eq. 2, based on the scale of environmental values, was set to 0.01. The strength of the species-environment feedback, α, which is the degree to which it could alter the environment towards the species optimum and away from the environment's baseline state, varied between 0 and 1.0 at intervals of 0.1 units. When there was no immigration, we calculated limiting similarity, which is the average distance between adjacent species' niche optima. We simulated 20 replicates for each set of parameter values, which results in 5280 simulations (20 replicates * 11 values of relative strength of the feedback * 4 levels of dispersal scale * 3 levels of immigration rate * 2 types of baseline environments). Each simulation ran for 100 000 generations; our simulations showed stabilization of species richness after 30 000 generations.

The above set of factorial simulation experiments is the focus of our study. To understand our results in a broader context, however, we relaxed some critical assumptions regarding symmetry of the species' engineering trait, priority effect and spatial configuration of heterogeneity. To avoid making our presentation overly complex, we leave a complete description of the additional simulations and results to Appendix A. Here we provide only an overview of the three relaxed assumptions. (1) In the factorial simulated scenarios described above, we assumed symmetry of the species' engineering trait; that is, all species had the same *α* value. In Appendix A, we relaxed this assumption by assigning *α* values randomly to the species between 0 and 1.0. (2) In the factorial simulated scenarios, we assumed best local competitors had prior arrival at a given cell. In Appendix A, we relaxed this assumption by random initial occupancy, in which all species had equal probability to occupy any grid cell before simulation starts. (3) In the factorial simulated scenarios, we assumed that the baseline environment is either homogeneous or heterogeneous in the form of a gradient. In Appendix A, we investigated a third spatial configuration of baseline environment, which is a heterogeneous random environment. We assigned the baseline environment *E*_*0*_ randomly between 0 and 1.0, as described in Appendix A.

## Results

### Species distribution and niche modification

**Figure 1:**
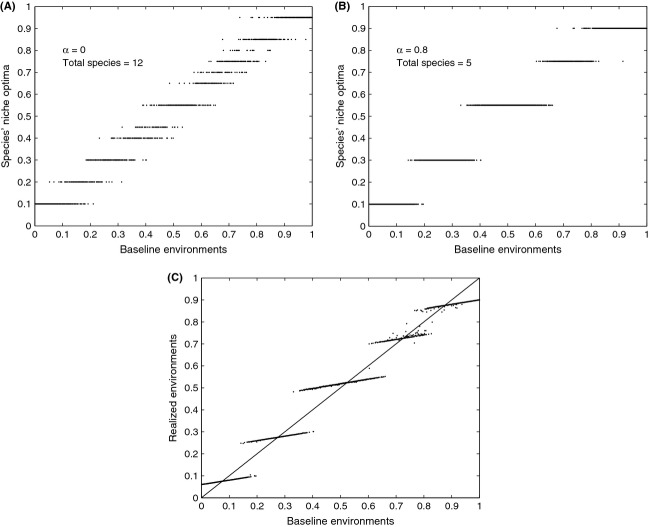
Distribution of niche optima of all individuals on a one-dimensional landscape (A) without species-environment feedback; (B) with relative strength of the feedback, *α =* 0.8 and (C) realized environmental condition, *E*_x_, along baseline environmental gradient, where the straight diagonal line represents the environment value without any change by feedback from species. For these runs, *m =* 0, *J =* 1000, *S =* 20, *b =* 0.05, *d =* 20.

**Figure 2:**
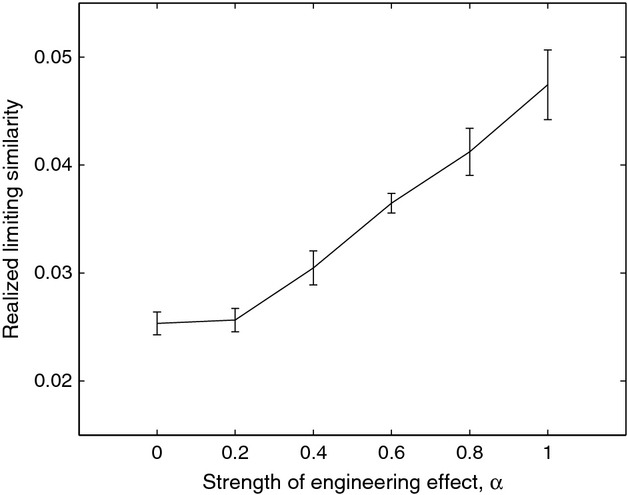
Realized limiting similarity as a function of relative strength of the species-environment feedback, *α*. Error bars indicate standard deviations over 20 replicate runs. For these runs, *m* = 0*, J* = 1000*, S* = 100*, b* = 0.01*, d* = 20.

**Figure 3:**
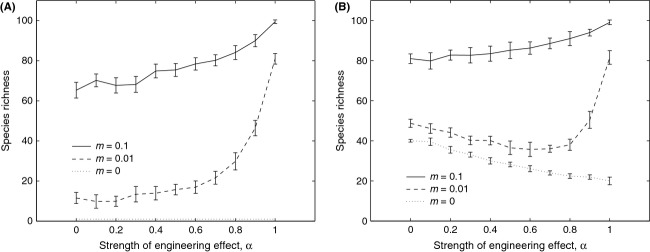
Species richness changes with relative strength of the species-environment feedback, *α*, under (A) homogeneous environment and (B) heterogeneous gradient environment. Error bars indicate standard deviations over 20 replicate runs. For these runs, *m =* 0*,* 0.01 or 0.1*, J =* 1000*, S =* 100*, b =* 0.01*, d =* 20.

We first demonstrate how the engineering affects spatial structure along the heterogeneous gradient, without the effects of immigration. When the one-dimensional landscape was assumed to have environmental heterogeneity in the form of a linear gradient along the spatial axis for the case in which there is no immigration (*m* = 0), the species sorted out on the landscape according to their niche optima and there was relatively even spacing between coexisting species (Fig. 1, Fig. S1). Note that Fig. 1 shows a case with *S* = 20, less than original simulation set up (Fig. S1), in which *S* = 100, to better display the resolution of the slope (Fig. 1C, Fig. S1C). For the rest of this article we used the parameter values introduced in the Simulation section. Realized limiting similarity, or the average distance between coexisting species' niche optima, increased with relative strength of the species-environment feedback (compare Fig. 1A and Fig. 1B). Fig. 2 plots limiting similarity against a sequence of values of α from 0 to 1, showing a positive relationship between limiting similarity and engineering trait strength, which results in a negative relationship between species richness and engineering trait strength (Fig. 3B, *m =* 0). Within each of the segments dominated by a species, the slope of the environment was displaced from the original baseline slope to a slope of approximately 1*- α* (Fig. 1C).

### Species richness and community structure

We expand the results to include the effects of immigration on both the homogeneous and heterogeneous gradient environments. Under a homogeneous baseline environment (*E*_0_ = 0.5), species-environment feedback created environment heterogeneity, thus facilitating diversity, unless there was no immigration, in which case only one species dominated, that species which is the best local competitor for the environmental condition 0.5 (Fig. 3A). Under a heterogeneous gradient environment, the initially negative relationship between species richness and strength of feedback, α, (the diversity-feedback relationship), shifted to a positive relationship when the immigration rate was increased from *m* = 0 to 0.01 and 0.1 (Fig. 3B). When the immigration rate was zero, increasing the species-environment feedback from the engineers, α, decreased species richness. At the low immigration rate (*m* = 0.01), species richness initially declined with increasing α, but further increases in α (α > 0.8) led to a sharp increase in diversity. At the high immigration rate (*m* = 0.1), the relationship between α and diversity was monotonically positive.

**Figure 4:**
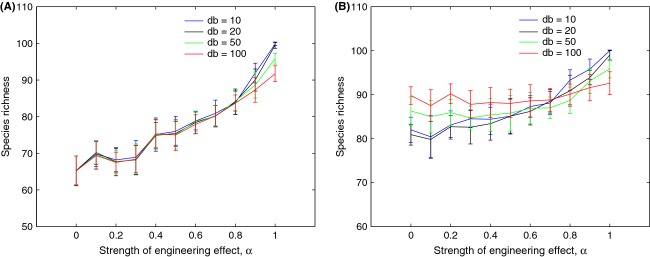
Species richness changes with relative strength of the species-environment feedback, *α*, when immigration rate was high, *m* = 0.1, under, (A) homogeneous environment and (B) heterogeneous environment. Error bars indicate standard deviations over 20 replicate runs. For these runs, *d =* 10, 20, 50 or 100*, J =* 1000*, S =* 100*, b =* 0.01, *m* = 0.1.

Dispersal scale, *d*, can also change the pattern of the diversity-feedback relationship. In the absence of immigration, or for a low immigration rate (*m* = 0.01), a larger dispersal scale (larger *d*) reduced species richness (Fig. S2), which, as in Fig. 3, also varies with increasing α. However, dispersal scale had little effect on the diversity-feedback relationship when there was high immigration rate (*m* = 0.1), under a homogeneous baseline environment (Fig. 4A). Interestingly, when immigration rate was high and heterogeneity existed in the form of a gradient in environmental conditions, the relationship between dispersal scales, *d*, and species richness were opposite at opposite ends of the species-environment feedback axis (Fig. 4B). When the feedback, α, was low or in the absence of feedback, species richness increased with dispersal scale, as other research has suggested (see review in Cadotte 2006). In contrast, when α was high, species richness decreased with *d* (Fig. 4B).

Our additional simulations (Appendix A) show that neither departures from the symmetry of species' engineering trait nor departures from the initial species distribution changed the pattern of our results for the diversity-feedback relationship qualitatively (Figs A1 and A2). Also, under a heterogeneous random environment, species richness was only weakly dependent on the species-environment feedback strength, but had a generally declining trend with the feedback (Fig. A3).

## Discussion

Our modeling framework for species-environment feedbacks in shaping community assembly suggests three important implications. First, community diversity responds differentially to engineering traits of species under different levels of immigration rate. For the heterogeneous gradient baseline environment, in the absence of immigration, species richness is negatively related to increasing feedback between species and environment, α. At low immigration rate, *m* = 0.01, species richness is negatively related to α when α starts from low values, but sharply increases at high values of α. When immigration rate is high (*m* = 0.1), species richness starts at a much higher value than for *m* = 0 and *m* = 0.01, at α = 0, and increases with increasing α over its whole range (Fig. 3B). For the homogeneous baseline environment, species richness increases with increasing α monotonically for both low and high immigration, and is also much higher for high immigration over the whole range of α. Very strong feedback always facilitates species richness in the presence of immigration from a regional pool, even though the immigration rate might be low. The reason is that, in the absence of immigration, local dispersal allows engineers through strong feedback to disperse to and modify nearby cells, thus expanding their ranges and decreasing overall species richness. When immigration is present (especially when high) on the other hand, it is more likely that species better suited for particular sites will reach those sites first and preempt those species that would otherwise have spread to and dominated the sites through engineering.

Second, the strength of engineering, α, shifts the pattern of diversity response to local dispersal. In the traditional niche-structured community theory, each species is the absolute best competitor in some segments of the niche axis. Without dispersal limitation, that is, when species disperse broadly, each species would be expected to ultimately reach and dominate the sites for which it is the best competitor. When the scale of dispersal is strongly limited in range, many species will not be able to reach suitable sites (Hurtt and Pacala 1995). This limits diversity, and, therefore, species richness increases with dispersal scale. However, these niche-structured community assembly theories, which involve the roles of niche and neutral processes in shaping community structure, do not include effects of ecological engineers (Tilman 2004; Gravel et al. 2006). When engineers are present and their species-environment feedback effects are strong, along with a high immigration rate, we found that species richness response to local dispersal range, *d*, can shift to a negative relationship when α is high. In that case, increasing dispersal decreases species richness (Fig. 4B for α ≍ 1). This is because, when the engineering effect is strong, a high range of dispersal favors engineers spreading rapidly and modifying the sites in which they settle to favor themselves before the sites can be reached and settled by species better suited to the baseline environment. Therefore, the relationship between species richness and α is reversed from the case when engineering strength is small (Fig. 4B for α ≍ 0). When the baseline environment is homogeneous and immigration is high, large α, as noted above, favors high species richness, but dispersal range has little effect because in the homogeneous case there are initially few favorable sites for the majority of immigrants, so differences in dispersal have little effect (Fig. 4A).

Third, environmental heterogeneity impacts the interactions among species-environment feedback, immigration and local dispersal scale. When the baseline environment is homogeneous, invasions by species from the regional species pool can create environmental heterogeneity through their engineering activities, thus increasing diversity. But to do this, the invaders must be able to preempt sites and modify them through engineering before the one species best suited to the homogeneous environment, e.g. for *μ*_*i*_ = 0.5, becomes established throughout (Fig. 3A). When the baseline environment has a linear gradient along the spatial axis, the relationship between species-environment feedback and species richness is more complex, because under certain circumstances species richness can decline with increasing feedback strength. For relatively low range of dispersal (*d* = 20, as in Fig. 3B) immigration favors the best local competitors expanding their space along the spatial axis via engineering activities, while constraining other invading species when the immigration rate is low (*m* = 0.01). In this case, only if the species-environment feedback is very strong (α ≍ 1), does the local community experience more neutral processes, allowing higher species richness, because the local environmental states are more likely to be exchanged back and forth by new immigrants. When the baseline environment has a random configuration (Appendix A), species are more likely to access unsuitable habitat through source-sink dynamics. In this case, species' engineering effects have weaker impacts on local species richness, but still have a declining trend with increasing strength of species-environment feedback when immigration is low or zero (Figs 3A,B,C).

Our model provides theoretical implications regarding the effects of ecological engineers on community diversity. The debate on what is the nature of the engineering-diversity relationship has lasted for two decades, since the term ‘ecosystem engineer’ was introduced (Jones et al. 1994; Wright et al. 2006). Some studies indicated that engineers increased species richness, if they increased environmental heterogeneity by altering habitat structure and distributions of resources (Wright et al. 2006). The effects of ecosystem engineering on species richness was also said to vary depending on the productivity of the system and whether engineers increase or decrease productivity (Wright and Jones 2004). In other studies, engineers were asserted, alternatively, to either increase or decrease diversity by changing habitat complexity (Crooks 2002). The results presented here represent an explicit elaboration of the degree of engineering effects on species richness, following the suggestions of Hastings et al. (2007) that the importance of ecosystem engineering is a matter of degree and scale. In the presence of engineering effect, α, with moderate strength, we found that this feedback enhances interspecific competition. Each species tends to expand its spatial extent through altering the baseline environment of whichever spatial cells it reaches towards its optimal environmental conditions. Higher competition squeezes out inferior competitors, consistent with limiting similarity, in which there exists a maximum level of niche overlap between competing species that will allow these species to coexist (Abrams 1983). However, when species-environment feedbacks are very strong (α ≍ 1) and/or the immigration rate is high, more species have a chance to invade and rapidly alter their local environment in their favor. This leads to finer-scale zonation along the environmental gradient. The range of of dispersal also plays a role. When species-environment feedback is strong, dispersal limitation (small *d*) favors many species creating local conditions favorable to themselves along the environmental gradient and surviving over the long term (Fig. 4B, *d* = 10 or 20).

Our model suggests that the direction of engineering-diversity relationship depends on both the immigration rate and the scale of local dispersal. Future studies on community-level effects of engineers should take into account immigration rate and local dispersal, as well as the strength of the engineering effects.

## Appendix A

Simulating random engineering strength, random initial species distribution, and random environment spatial configuration.

To place our results in a broader context and understand them better, we relaxed some critical assumptions regarding symmetry of the species' engineering trait, priority effect and spatial configuration of heterogeneity. In the main text simulations, we assumed that (1) species' engineering trait were symmetric; that is, all species had the same strength of species-environment feedback, α; (2) the best local competitor occupied each site of the landscape initially; (3) in the case of a heterogeneous environment, the landscape was in the form of a monotonic gradient. Here, we provide some additional simulations when these assumptions are relaxed.

### Simulations and Results

#### Randomly assigned α

Each species in the regional pool was assigned randomly a trait value of engineering strength, α; that is, the strength of species-environment feedback, α, was randomly distributed around 3 levels of average traits, 0.25, 0.5 and 0.75, for the two cases of no immigration and immigration rate *m =* 0.1. When the mean (α) *=* 0.5, all the species' engineering trait was a uniform distribution between 0 and 1.0. Analogously, species' engineering traits were uniformly distributed [0, 0.5] and [0.5, 1.0], for mean (α) *=* 0.25 and 0.75, respectively. We compared the results to Fig. 3B, which assumed symmetry of engineering traits under a heterogeneous gradient environment. Other parameters were the same as indicated in Fig. 3B.

When the assumption of symmetric engineering trait was relaxed, species richness was slightly lower compared to the same level of fixed α value in the symmetric case. But the diversity-feedback relationships did not change qualitatively (compare Fig. A1 with Fig. 3B).

#### Random initial assignments of species on the landscape

In this case, each species had equal probability of occupying any grid cell in the landscape. We did simulations similar to those in Fig. 3B, when immigration rate varied as *m =* 0, 0.01 and 0.1, under a heterogeneous gradient environment. We also investigated the impacts of the initial distribution under a heterogeneous random environment. Information is provided below for how the random environment was configured.

When the assumption that each grid cell was initially occupied by the best local competitor was relaxed, such that all the species randomly occupied the landscape initially, the results also did not differ qualitatively (compare Fig. A2 with Fig. 3). In the case of high immigration rate, diversity-feedback relationships were almost the same between the two initial occupancy assumptions, both under the gradient and random environments. In the absence of immigration, species richness was much lower when species randomly occupied the landscape compared to the case in which the best local competitor occupied the landscape, when the landscape was a heterogeneous random environment (Fig. A1b).

#### Heterogeneous random environment

We investigated how the species-environment feedback affects local species diversity under a heterogeneous random environment. In this case, each lattice cell was assigned a random baseline environmental value between 0 and 1.0. A full factorial set of simulations of three levels of immigration rate, *m* = 0, 0.01 and 0.1, and three levels of local dispersal scale, *d* = 20, 50, and 100, were investigated. Other parameters were the same as indicated in main text.

Under the heterogeneous random environment, the species richness showed independence or only a weak dependent relationship to the species-environment feedback, but still had a declining trend with increasing strength of species-environment feedback (Fig. A3). In this scenario, local dispersal scale had no effect on species richness when immigration rate was high, *m* = 0.1 (Fig. A3a). Species richness declined as local dispersal scale increased; that is dispersal became less limited, when immigration rate was low (Fig. A3b) or in the absence of immigration (Fig. A3c). The overall species richness was higher than for the gradient environment (compare Fig. A3 with Fig. 3).

## Supporting Information

**Figure S1.** Distribution of niche optima of all individuals on a one-dimensional landscape a) without species-environment feedback; b) with relative strength of the feedback, α = 0.8 and c) realized environments along baseline environmental gradient, where the straight diagonal line represents the environment value without any change by engineering feedback from species.

**Figure S2.** Species richness changes with relative strength of the species-environment feedback, α, when a) absent of immigration, *m* = 0, under heterogeneous gradient environment, b) low immigration rate, *m* = 0.01, under heterogeneous gradient environment and c) low immigration rate, *m* = 0.01, under homogeneous environment.

**Figure A1.** Species richness changes with relative strength of the specie-environment feedback, α, when immigration rate *m* = 0, 0.01 or 0.1 (same figure as Fig. 3B except the red circles).

**Figure A2.** Comparison of impacts of initial species distribution on diversity-feedback relationship, between the case in which all the local were initially occupied by best local competitor (bold lines) and the case in which random occupancy was assumed (thin lines), under a) heterogeneous gradient environment, and b) heterogeneous random environment.

**Figure A3.** Species richness changes with relative strength of the specie-environment feedback, α, in a heterogeneous random environment, when a) immigration rate *m* = 0.1; b) immigration rate *m* = 0.01; and c) in the absence of immigration, *m* = 0.
