# Supplementary material for: Strong species-environment feedback shapes plant community assembly along environmental gradients
Source: Ecol Evol. 2013 Sep 20;3(12):4119–28. doi: 10.1002/ece3.784 (PMC3853557; doi:10.1002/ece3.784)
Supplement: Supplementary file 13 [file ece30003-4119-SD13.docx]

Description of Supplemental materials

**Fig. S1.** Distribution of niche optima of all individuals on a one-dimensional landscape a) without species-environment feedback; b) with relative strength of the feedback, *α =*0.8 and c) realized environments along baseline environmental gradient, where the straight diagonal line represents the environment value without any change by engineering feedback from species. For these runs, *m =*0, *J =*1000, *S =*100, *b =*0.01, *d =* 20.

**Fig. S2.** Species richness changes with relative strength of the species-environment feedback, *α*, when a) absent of immigration, *m* = 0, under heterogeneous gradient environment, b) low immigration rate, *m* = 0.01, under heterogeneous gradient environment and c) low immigration rate, *m* = 0.01, under homogeneous environment. Error bars indicate standard deviations over 20 replicate runs. For these runs, *d =*10, 20, 50 or 100*, J =*1000*, S =*100*, b =*0.01*.*
